# Inline Analysis of the Plastics Melt Emissions in Real Time During Mechanical Recycling Using the SIFT-MS Method

**DOI:** 10.1021/acsomega.5c08107

**Published:** 2025-12-12

**Authors:** Felix Mehrens, Niklas Rode, Jacek Lecinski, Madina Shamsuyeva, Hans-Josef Endres, David Müller

**Affiliations:** † IKK - Institute of Plastics and Circular Economy, 26555 Leibniz University Hanover, An der Universität 2, 30823 Garbsen, Germany; ‡ Syft Technologies Limited, 68 St Asaph Street, Christchurch Central, Christchurch 8011, New Zealand

## Abstract

The quality of recycled plastics is significantly influenced by the contamination content of volatile organic compounds (VOCs), which enter the plastic through additives, migration or degradation processes. This poses a challenge for applications with high chemical requirements, such as food and cosmetics packaging or components in automotive interiors. In addition, previous analytical methods are based on random samples and only analyze a fraction of the material. Particularly with inhomogeneous input streams, there is a risk that potentially harmful substances will not be detected. For this reason, this study investigated the suitability of Selected Ion Flow Tube Mass Spectrometry (SIFT-MS) for the continuous inline analysis of VOCs during the extrusion process. By measuring the gas flow in real time at a vacuum dome of a twin-screw extruder, various VOCs from the input materials, including limonene and model substances in accordance with FDA requirements, were clearly identified and quantified. The measurement results showed a high correlation between the VOC content in the input material and the concentration in the gas flow. The results demonstrate the potential of SIFT-MS for process monitoring and quality control in mechanical plastics processing, especially for increasing safety and traceability when using recycled materials in sensitive applications.

## Graphical abstract

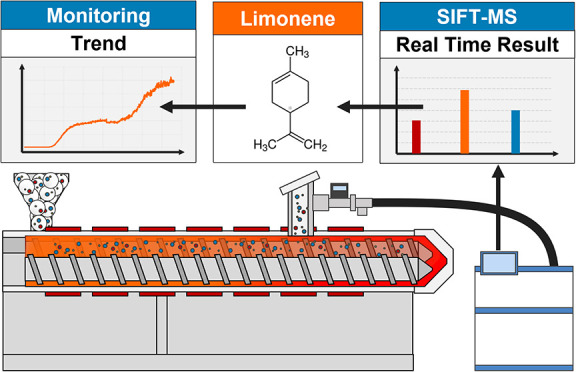

## 1 Introduction

Plastic products can be found in almost all areas of everyday life and in industry today. Thanks to their flexible, adjustable properties, low price and ease of manufacture, their areas of application vary from use in various packaging products to use in technical applications such as automotive, electrical and electronic systems, etc., thus increasingly substituting other materials. This is also reflected in the steadily increasing annual plastic production, which has increased from 245 million tons in 2008 to 413.8 million tons in 2023.^1^ The majority of produced plastics, at 44%, are used to manufacture packaging products with a typical lifespan of less than one year.^2,3^ The directly related increase in the amount of plastic waste shows the importance of the end-of-life treatment of these products.^4^ Accordingly, forecasts indicate that the amount of plastic waste will increase from approximately 155.87 million tons in 2024 to 379.97 million tons in 2060.^5^ Today, the majority of plastic waste is sent to landfill (46%) or incinerated (17%), leading to pollution and the release of greenhouse gases.^6^ For this reason, the aim is to move toward a circular economy in which plastic can be reused through recycling at the end of its life cycle. Recycled materials therefore offer the opportunity to reduce the consumption of fossil resource while at the same time reducing the environmental impact and influencing factors of plastic products.^7^ To achieve this, mechanical recycling has been established in industry as a process for producing recyclates from plastic waste, in which the shredded plastic is melted, filtered, degassed and then regranulated.^8–10^

But so far, the recycling rate of plastic waste is currently only at 9% worldwide.^11^ Although the recycling rate in Europe is significantly higher at 40.7% in 2022 for plastic packaging, the largest quantities of this are downcycled into products with low material requirements.^12–15^ This is mainly because the quality of the recycled material often does not meet the requirements for the material. In addition to degradation, which primarily leads to a deterioration in the mechanical and thermal properties, it is the contamination in the plastic that prevents its use in many areas with specific requirements in terms of chemical quality.^16^ A large part of these contaminants consists of volatile organic compounds (VOCs). These substances either migrate into the plastic from contact products during its life cycle, form within the plastic as degradation products of the polymer, or are added as additives.^17^ If these substances are not removed during the recycling process, they can migrate from the recycled material back into the ambient air or a contact material.^18^ A large number of studies and reviews deal with the analysis and identification of these substances in postconsumer recyclates (PCR).^18–20^

At the same time, ensuring the chemical quality of plastics and recycled materials used in contact-sensitive applications poses a particular challenge. In particular, in areas such as food or cosmetics packaging and automotive interiors, legal or industry-specific regulations apply to the chemical composition and safety of the materials used. For example, the use of plastics intended to come into contact with food is regulated by European Union Regulation 10/2011 ^21^ which sets out the requirements for plastics in general, and Regulation 2022/1616,^22^ which sets out explicit requirements for recycled plastics. Among other things, these requirements include maximum limits for a variety of substances that may be present in plastic or recyclate. For the use of plastics and recyclates in other applications, such as cosmetic packaging and automotive interiors, the regulatory requirements are less specific. In accordance with the REACH regulation, these are based on the personal responsibility of the industry and the distributors of products. Initial approaches to an optimized regulation were summarized in the CosPaTox project as a voluntary guideline for industry on the safe use of recycled postconsumer plastic in cosmetic packaging,^23^ which was subsequently converted into a German standard DIN SPEC 91521. While these two packaging applications have requirements for the specific chemical composition of the plastic used, the focus for automotive interior components is on the total emissions of the volatile and semivolatile components of the plastic or recyclate without specific evaluation of the toxicological properties. Typical emission limits are 250 μg/g of plastic for VOCs and 500 μg/g for low volatile organic components according to the VDA 278 measurement method.^24^

This leads to two essential requirements for the recycling process. On the one hand, all these volatile contaminations must be removed up to the applicable limit values in the recycling process and, at the same time, this must be monitored using suitable analytical methods.^19,25,26^ This applies particularly to inhomogeneous recyclate input streams from open-loop collections, such as the “yellow bin” in Germany, in which packaging from a variety of different applications with a wide range of contact materials ends up. Accordingly, the amount of different contaminants in the recyclate input stream is large, as the studies mentioned above have shown.^19^

State of the art analytical methods for identifying the VOCs in a plastic sample are based on gas chromatography–mass spectrometry (GC-MS), in which the volatile substances are separated in the chromatography column and then are identified by their mass-to-charge ratio (*m*/*z*-ratio) in the mass spectrometer.^17,27^ The analysis methods differ mainly in the type of sample preparation used to extract the volatile substances from the polymer. Due to the minimal effort involved in terms of automation, headspace methods have become particularly prevalent here. In these methods, the volatile substances are transferred into the gas phase by heating the plastic samples and transported directly into the gas chromatography column.^27–29^

Currently, the chemical composition of a recyclate is typically determined by analyzing random samples in specialized laboratories, shown in Figure 1. In the process, only a few grams of a batch of several tons are tested. This creates the risk that the analysis will not represent the actual composition of the batch, meaning that potentially harmful substances which are not present in the tested sample may remain undetected in the batch. Therefore, this lack of continuous monitoring carries the risk that contaminations will not be detected, meaning that contaminated recyclates could be used in critical application areas and pose a risk to consumers of products packaged in them.^26,30^

**Figure 1:**
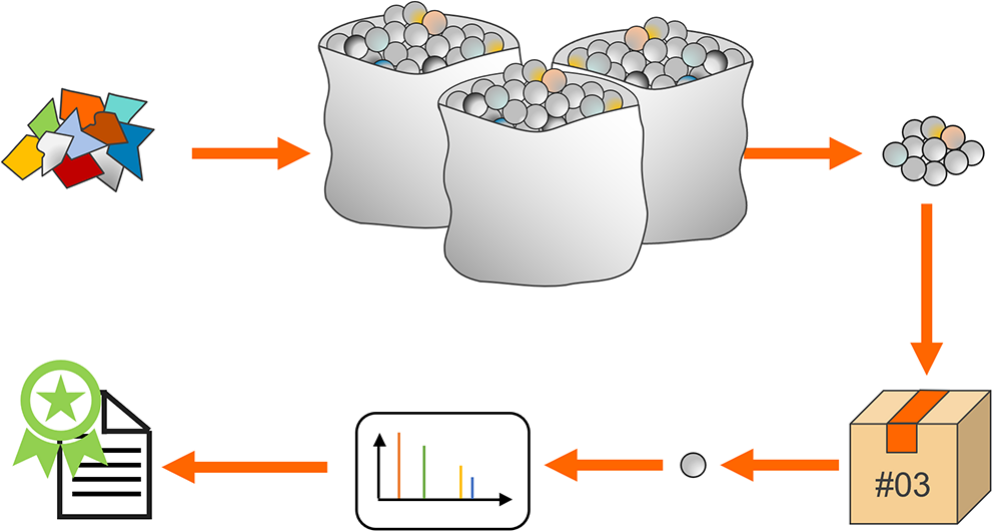
Schematic representation of the state of the art in recyclate analysis.

Consequently, there is a great demand for the development of methods testing the chemical composition of an entire batch of plastic recyclate with a detection limit at least comparable to GC-MS or better. For this reason, continuous monitoring of plastic quality during extrusion is necessary to ensure safe use of recyclates from inhomogeneous input streams. A promising solution is the real-time monitoring of the chemical quality by the online analysis of VOCs using the SIFT-MS technique (Selected Ion Flow Tube Mass Spectrometry) from a gas sample.^31^ Due to the high temperatures in the extrusion process, the volatile substances diffuse into the gas phase and can be analyzed there, analogous to a headspace, if they are extracted. This method enables volatile substances such as VOCs, which tend to migrate, to be detected and analyzed directly in recycling process. This offers particular advantages here: it allows a continuous analysis of VOCs in real time.^32^ Investigations by Langford and Perkins^33^ showed the successful application of a SIFT-MS for use in analyzing the headspace of various recyclates and virgin polymers. This makes it a suitable method for continuously monitoring the chemical quality of recyclates in real time, as well as for evaluating both decontamination measures and the homogeneity of the material.^34^

This study aims to investigate the potential applications of SIFT-MS for measuring volatile substances in real time in the gas stream during the extrusion process. For this purpose, various materials with different types of contamination, such as limonene, were tested for VOCs. Limonene is considered a model compound because it is frequently found in recyclates and is a typical representative of VOCs.^19,35,36^ The results should demonstrate how this technology can enhance the quality of recycled materials, thereby meeting the requirements for sustainable, resource-efficient plastic processing.

## 2 Material and Methods

### 2.1 Sample Description

For this study, the polypropylene (PP) PPH 750 (TotalEnergies, France) with a melt flow index of 12 g/min (2.16 kg/230 °C) was contaminated with DL-limonene (≥95%, Merck, Germany) and ethanol (≥98%, Carl Roth, Germany) as a carrier solvent. For the contamination, 50 kg of the PP were placed in a 200 L barrel together with 1 L of ethanol and 100 mL of D-limonene. The barrel was then sealed and heated isothermally at a temperature of 40 °C using a barrel heater and stored for 21 days with daily agitation. After that, the barrel was opened for 1 day to allow the residual solution to evaporate.

The high-density polyethylene (HDPE) used for the trials was kindly supplied by KraussMaffei Extrusion (Hannover, Germany). This material was contaminated according to the US Food and Drug Administration (FDA) requirements (FDA-Material)^26,37^ for a challenge test to approve recyclates for use in food packaging. In accordance with FDA requirements, the following substances were used as model substances: chlorobenzene for the polar volatile category, toluene for the polar nonvolatile category, methyl salicylate and benzophenone for the nonpolar volatile category, methyl stearate and cyclohexylbenzene for the nonpolar nonvolatile category.

Ground and washed PP flakes from mixed postconsumer packaging waste collected via the ‘yellow bin’ collection system in Germany with the material specification 324-0 (DSD-324) were purchased at the market.

### 2.2 Selected Ion Flow Tube Mass Spectrometry

SIFT-MS is based on the developments for the SIFT technique by Adams and Smith.^38^ A schematic representation of the measurement principle of the SIFT-MS method is shown in Figure 2 and is explained below.

**Figure 2:**
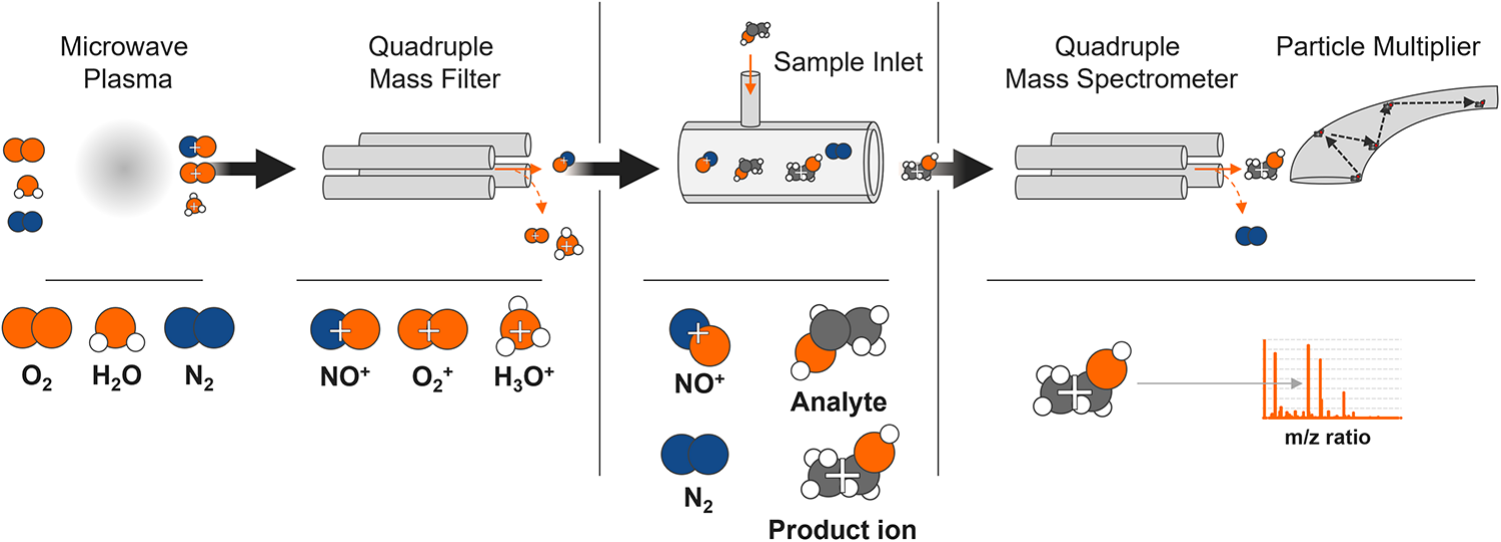
Schematic diagrams of a selected ion flow tube-mass spectrometer instruments in reference to Syft Technologies.

In the first step, the reagent ions are produced by microwave discharge in a microwave plasma in a humid atmosphere. The ions are usually the cations H_3_O^+^, NO^+^ and O_2_^+^ or the anions O^–^, O_2_^–^, OH^–^, NO_2_^–^ and NO_3_^–^. The advantage of these ions is that they rarely react with the components present in large numbers in the air, such as nitrogen or oxygen, but do react with a large proportion of the trace gases in a gas sample. The reagent ions are extracted from the ion source and the desired ions are filtered by a quadrupole mass filter and passed into the carrier gas, which is either helium or nitrogen. In the flow tube, the reagent ions then react with the VOC molecules from the introduced gas sample, resulting in the formation of the analyte ions. Subsequently, a downstream quadrupole mass spectrometer (QMS) and a particle multiplier are used to detect, identify and count the *m*/*z*-ratio of the product ions. Based on this information, the concentrations of each neutral analyte in the sample quantity can be calculated.^31,39,40^

The measurements can be carried out in a full-scan (FS) or selected ion mode (SIM). In the full scan mode, the entire spectrum of *m*/*z*-ratios of the ions is recorded, whereas in the selected ion mode, the QMS downstream of the flow tube acts as a filter, removing ions outside a defined *m*/*z* range. Analyzing only the product ions within the defined *m*/*z* range simplifies data evaluation and reduces the measurement time.^31,41^

### 2.3 Diffusion of VOCs from the Polymer Melt

In order to analyze the VOCs from the input material in the extrusion process using the SIFT-MS method, they must be transferred from the polymer melt to the gas phase. The mass transfer of the VOCs from the melt into the gas phase in the extruder takes place by equilibrium-controlled diffusion and is mainly based on two mechanisms.^42^ One is the concentration difference at the direct surface of the melt to the gas phase at the venting ports. As soon as the pressure of the vapor pressure of the volatile substance exceeds the gas phase pressure, it diffuses through the polymer-gas interface into the gas phase. Due to the constant renewal caused by the rotating movement of the screws, the concentration gradient is constantly renewed and thus a continuous mass transfer of the VOCs from the melt into the gas phase takes place. The second mechanism is the formation of bubbles of volatile substances in the polymer melt under certain conditions. These grow, merge and reach the interface between the polymer and gas phase, where they burst and pass into the gas phase. The speed and quantity of the mass transfer depend not only on concentration equilibrium but also, among other things, on temperature, ambient pressure, vapor pressure, molecular weight and surface renewal rate.^43,44^ The influence of these extrusion process parameters on diffusion behavior has already been examined in a large number of practical and theoretical studies^45–48^ and was not systematically investigated in this work. At the same time, these relationships play an important role, as only the compounds and the quantities of compounds that are volatile under the conditions of the extrusion process can be analyzed.

### 2.4 Experimental Setup

To perform inline measurement of VOCs released from the polymer melt the Syft Tracer (Syft Technologies, Christchurch, New Zealand) was connected to a corotating twin-screw extruder (ZE28 Bluepower, KraussMaffei Extrusion) at the degassing port via a 2.5 m long, heated to 120 °C sample line, as Figure 3 shows. The schematic setup of the extruder and sampling process is shown in Figure 4. Two gravimetric feeders (DDW-M-FW40, Kubota Brabender Technologie GmbH, Germany) were used to ensure a constant combined feed rate of 16 kg/h at a fixed extruder screw speed of 150 rpm. Settings in the corotating extrusion process were kept constant for all tests in order to achieve direct comparability of the measurement results. The extruder used has a total length of 56 L/D and consists of 13 barrel sections with 4 or 6 D, each with its own heating element. The temperature profile is shown in Table 1.

**Figure 3:**
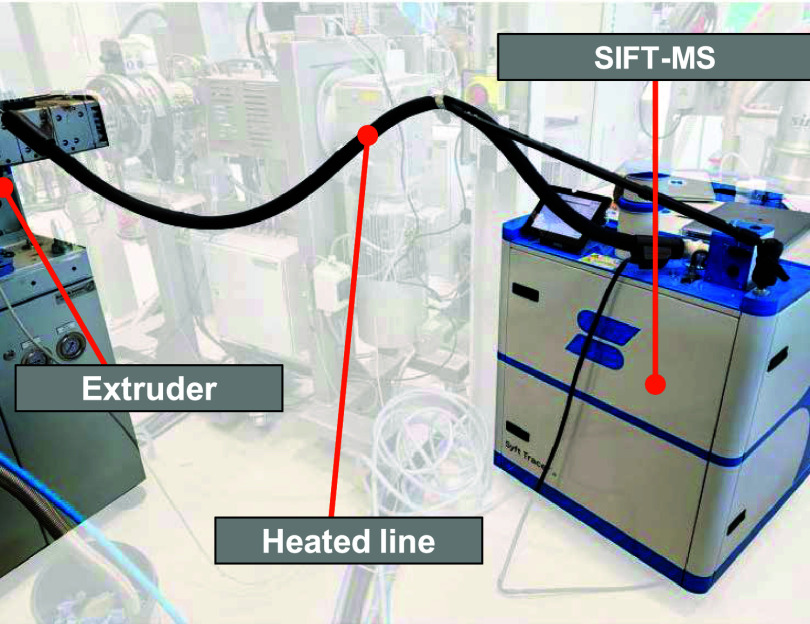
Illustration of the inline measuring setup on the extruder.

**Figure 4:**
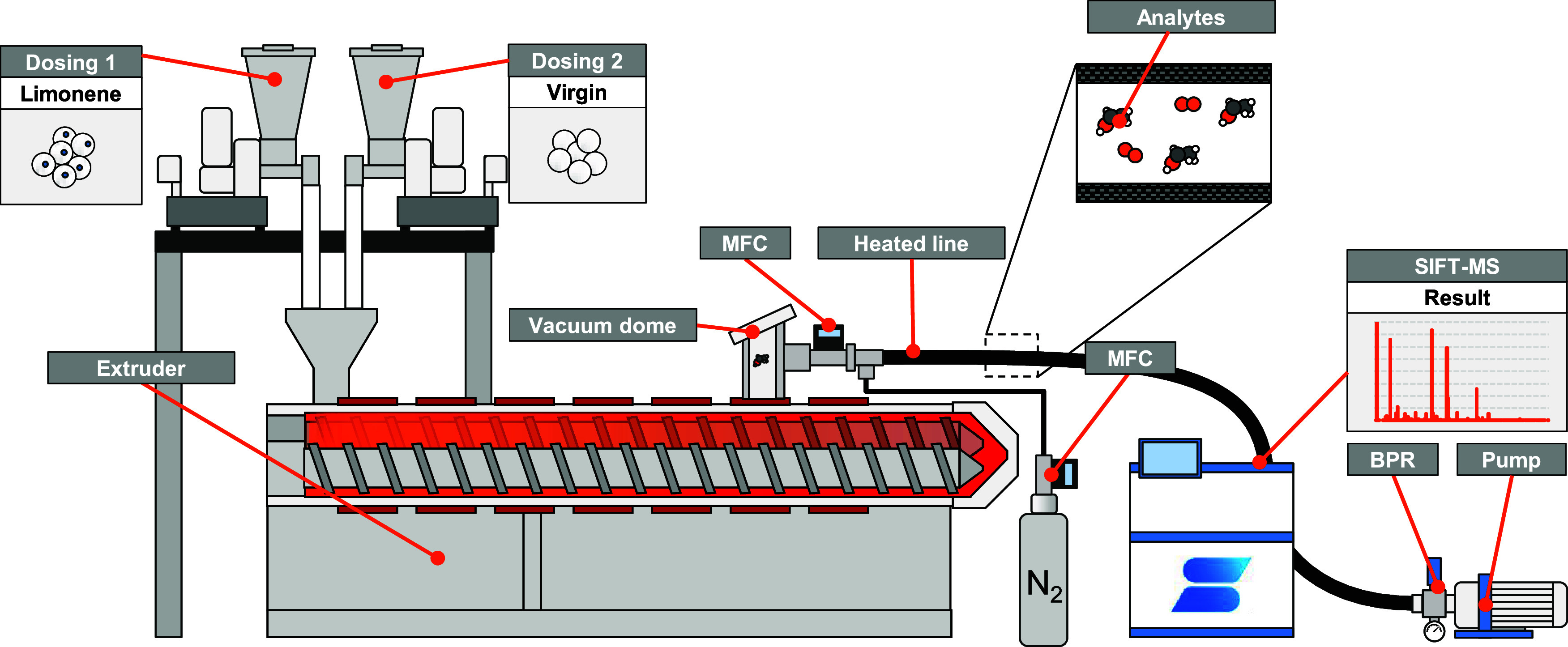
Illustration of the inline measuring setup on the extruder.

**Table 1:** Used Temperature Profile of the Extruder

temperature of the barrel sections [°C]						
1–2	3–4	5–6	7–8	9–10	11–12	die
170	180	190	200	205	210	220

The gas sample was taken from a vacuum dome on the 10th barrel of an open 6D housing element with a Type A insert. Screw elements with a low pitch were used in this area in order to achieve the largest possible melt surface and thus a high degassing performance. A pump with a back pressure regulator (BPR) set to 850 mbar was used to ensure a steady gas flow from the vacuum dome to the SIFT-MS inlet. The sample flow rate was set to 4 standard cubic centimeters per minute (sccm). Due to the high concentration of VOCs, the sample flow was diluted by adding nitrogen at a controlled rate of between 100 and 1100 sccm via a mass flow controller (MFC) (MCS series, Alicat Scientific), before entering the heated sampling line. This results in a dilution between 0.36 and 3.9%. The measurements were carried out using a continuous flow-past configuration, in which 25 sccm of the diluted gas stream were sampled for the analysis and introduced into the SIFT-MS inlet, while the remaining gas was exhausted. This was done to minimize sample condensation and ensure representative sampling.

For the investigation, FS and SIM measures were carried out in positive ion mode. Nitrogen was used as the carrier gas. The measurement data were obtained for the products of all three positively charged reagent ions (H3O^+^, NO^+^ and O2^+^) in the *m*/*z* range from 15 to 250 with unit mass resolution. The ion dwell time was 100 ms.

### 2.5 Methodical Approach

To determine the suitability of the Syft Tracer for the detection of impurities in the field of plastic recycling, three different materials were tested with three different research questions. First, the contamination of a material with limonene was used to investigate whether a corresponding quantitative analysis can occur in the gas sample chamber based on different concentration levels. Therefore, virgin PP and PP precontaminated with limonene were extruded and the sample gases released in the process were analyzed. To evaluate the quantitative determination of the amount of limonene contained in the sample gas, various compositions of the two materials were compounded according to the Table 2.

**Table 2:** Different PP- & PP-Limonene-Compounds

	Feeder 1	Feeder 2
	Virgin PP	Limonene PP
PP_Raw_	100%	0%
PP_L15_	85%	15%
PP_L25_	75%	25%
PP_L50_	50%	50%

In the second approach, the FDA-contaminated material was compounded with 65% virgin PP and the melt emissions analyzed to examine the measurability of a broad spectrum of chemical substances with different polarities, molecular weight and volatility levels.

In the third and final approach, PP flakes originating from postconsumer waste were extruded in two compositions (shown in Table 3) to validate the method for detecting unknown and untargeted VOCs present in the gas stream.

**Table 3:** Different PP- & Post-Consumer-PP-Compounds

	Feeder 1	Feeder 2
	Virgin PP	PCR–PP
PP_DSD15_	85%	15%
PP_DSD41_	59%	41%

## 3 Results and Discussion

Comparison of virgin PP and different limonene/virgin PP mixtures shows multiple peaks in SIM and FS measurements. Peaks that are present in all measurements most likely originated from short-chain molecules of PP, which enter the gas phase during processing. Measurements of the limonene-Virgin PP mixture show additional peaks that can clearly be assigned to limonene and ethanol, based on the *m*/*z*-ratio of their respective reagent ions. Table 4 shows an overview of the different *m*/*z*-ratios that correlate with the amount of limonene in the processed material. Due to the high ethanol concentration caused by the contamination process, a very large proportion of the reagent ions were already reacting with the ethanol molecules. Therefore, there are not enough ions left to react with the remaining molecules, such as limonene. This means that the quantification of ethanol and other substances is not valid and for this reason, the gas flow from the extruder had to be diluted by a factor of 1/250. By removing ethanol, which mostly adheres to the surface of the granules, e.g., by washing, lower quantities of limonene could have been measured more easily.

**Table 4:** Characteristic m/z-Ratios of the Different Reagent Ions That Correlate with the Concentration of Limonene in the Gas Phase

	ethanol			limonene		
Ion	H_3_O^+^	NO^+^	O_2_^+^	H_3_O^+^	NO^+^	O_2_^+^
*m*/*z*-ratio	47	45	44	81	92	68
	65	47	46	95	93	80
	93	63	65	137	94	92
		65	91		121	93
		91	93		135	107
		93			136	121
						136
						137

The reults of the SIM measurements of the different limonene and virgin blends showed that both marker substances can be clearly and easily detected in real time in a continuous extrusion process. The measured concentration for the product ion of NO^+^ with an *m*/*z*-ratio of 45 for ethanol and 136 for limonene is shown in Figure 5. It shows that 5 min after changing the dose to 15% of the limonene contaminated material (represented by the dotted line), there is an increase in the detected amount of limonene. A nearly constant limonene concentration around 0.35 to 0.4 ppm by volume (ppmV) is detected after 10 min. At the same time, it can be seen that the time taken for the initial detection and for the concentration level to reach a constant level varies for both substances. By contrast, ethanol has been detected after 2 min and reached saturation after 7 min. Due to the residence time in the extruder, a certain time delay between the change in the dosing rate and the first measurement is to be expected. The delayed detection shows the significant influence of molecular volatility (such as the lower vapor pressure and higher diffusion resistance of limonene) on the emission kinetics of various substances. Another explanation for the time difference is that limonene may have condensed in the heated line, which was only heated to 120 °C, well below limonene’s boiling point of 178 °C.

**Figure 5:**
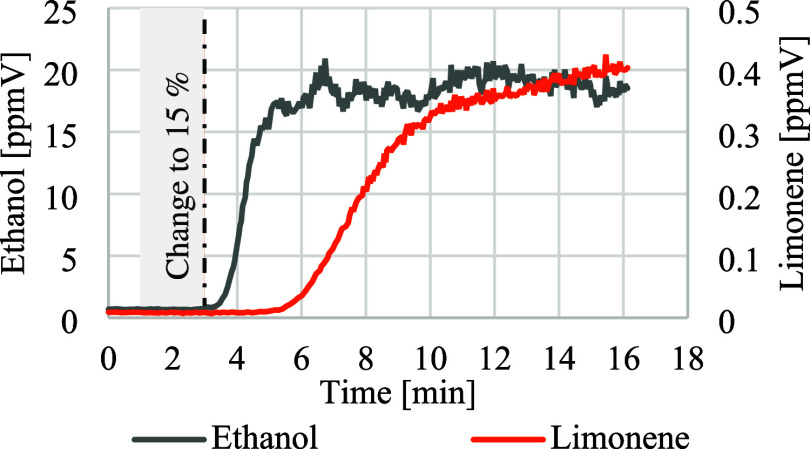
Curve of the detected limonene and ethanol concentration after the start of dosing.

The concentration also increases as a function of the amount present in the input stream to the extrusion process, as can be seen in Figure 6. The limonene concentration trend for the product ion of NO^+^ with a *m*/*z*-ratio of 136 clearly correlates with the dosed amount of contaminated material. After 18 min, the dosage setting is changed to 35% of the limonene. After about 5 min, the concentration increases again reaching a constant level of around 1 ppmV after 10 min. The results also show that deviations in VOC concentration at the sampling point are detected within seconds. The total detection time, from a change in polymer composition due to a change in the input material dosage to a change in the gas composition at the vacuum dome, is determined by the melt’s residence time in the extruder. This time can vary depending on the size of the extruder, the extrusion parameters and the throughput, and usually takes several minutes.

**Figure 6:**
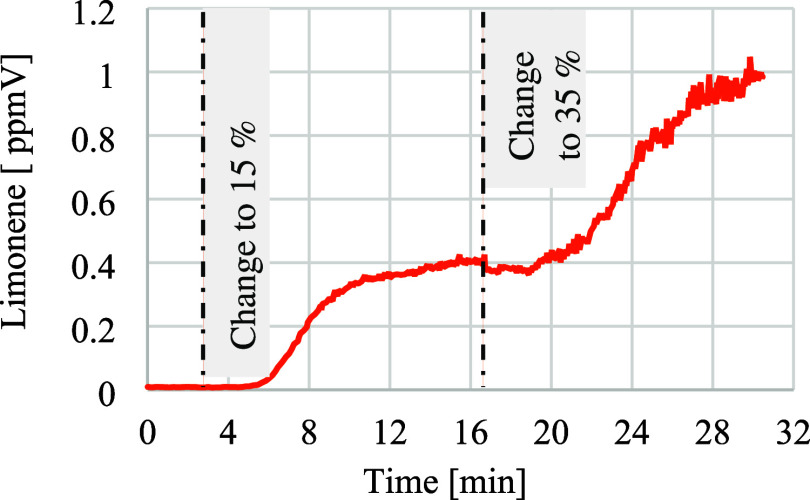
Limonene concentration in relation to the amount of material contaminated with limonene over time.

The SIFT-MS measurements show a high degree of linearity when only one product ion with *m*/*z* = 136 of the NO^+^ reagent ions is considered. Figure 7 shows the normalized signal at 10,000 counts per second (cps) per 1 million reagents, as well as limonene concentration in relation to the three material fractions contaminated with limonene. The coefficient of determination *R* ^2^ for the measured limonene signal is 0.997 and 0.9983 for the limonene concentration in the gas phase in relation to the amount of dosed limonene contaminated material.

**Figure 7:**
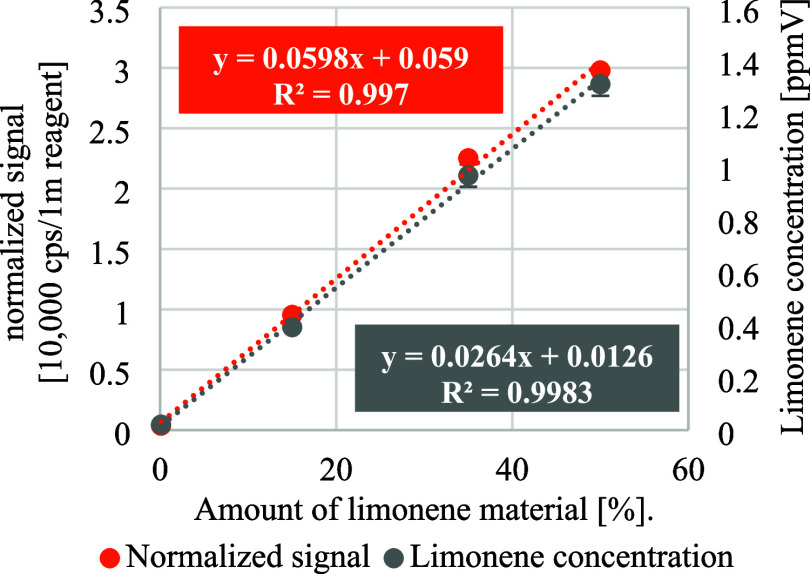
Normalized limonene signal and concentration in the gas phase in relation to the amount of contaminated material with limonene for *m*/*z* = 136 for NO^+^ as reagent ion.

These results demonstrate that, under the same conditions, limonene diffuses into the gas phase from the plastic melt in a highly linear manner with respect to the input amount and concentration. This applies to both the signal and the quantitative concentration in the gas stream. The linear correlation demonstrates the stability of the method when analyzing different concentrations of VOCs in the input material. However, to determine the exact input concentration in the material, further factors must be considered, such as volatility of a substance, pressure prevailing in the gas phase, temperature or concentration equilibrium.

When measuring the gas phase emissions from the FDA material, toluene, chlorobenzene, methyl salicylate and cyclohexyl benzene were all measurable with each reagent. At the same time, no benzophenone or methyl stearate could be detected in the gas phase. It should be noted that, due to the maximum chosen FS measuring range of 250 *m*/*z* in the measuring setup, it was not possible to measure methyl stearate with a molecular mass of 298.5 g/mol. The detection was not expected in general due to the properties of methyl stearates and the factors affecting the volatility given in the setup. This is mainly because the boiling point of methyl stearate is 448 °C at 1 atm, which is significantly higher than the 220 °C temperatures prevailing in the extrusion process. Figure 8 shows the H_3_O^+^ reagent ions signal in relation to the *m*/*z*-ratio from 15 to 250 for the FDA (gray) material and the uncontaminated virgin PP (orange). The signal detected for the substances decreases as their volatility decreases and their molecular weight increases. This corresponds with the expectation that the quantity of substances with low volatility diffusing from the melt into the gas phase during the extrusion process decreases. At the same time, it is possible that, again, a certain amount has already condensed due to boiling points above the temperature of 120 °C of the heated line from the vacuum port to the SIFT-MS. Increasing the melt temperature and raising the temperature of the heated line, while applying a vacuum, can increase the amount of the substance diffusing out of the melt by preventing condensation. The increase in diffusion from the melt is related to the methods used to remove VOCs from the melt and has been documented in various publications.^49–51^

**Figure 8:**
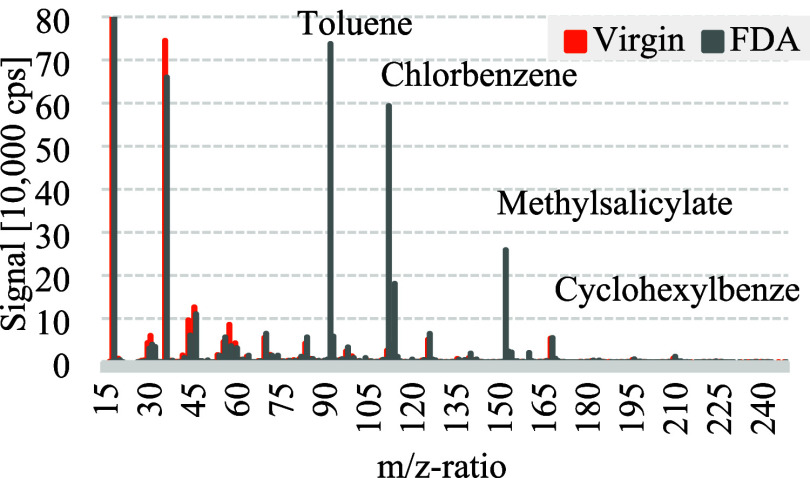
Detected signal of H_3_O^+^ reagent ions for the *m*/*z*-ratios for the virgin and FDA material.

Based on the measurements on the DSD-324 material, an increase in the total concentration in the gas phase of the emissions was measured. Therefore, the amount of the measured substances increases in line with the DSD-324 amount in the compound. The reagent ions reacted completely due to the high concentration of VOCs in the input material. Consequently, the dilution had to be increased in proportion to the percentage of DSD-324 in the input. The measurements showed a clear detection of limonene in the gas stream of the material flow. Figure 9 also illustrates this, showing the dilution corrected quantitative amount of limonene in the gas sample in relation to the proportion of recycled material in the throughput.

**Figure 9:**
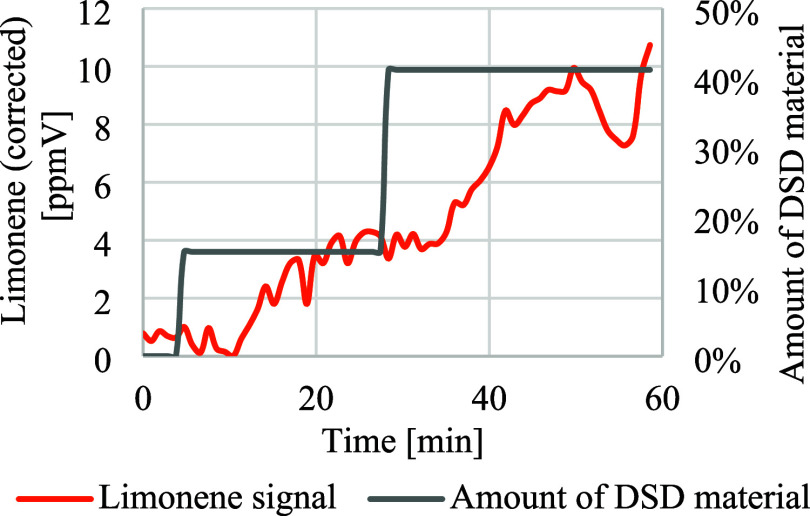
Total quantity of limonene measured in the gas phase (dilution corrected) in relation to the amount of DSD-324 material dosed.

The limonene concentration increases in proportion to the amount of postconsumer PP in the input material, comparable to the results obtained from deliberately contaminated limonene material. At the same time, the measurement results are subject to greater signal fluctuations, which can be attributed to the higher level of inhomogeneity in the input material. Another difference is that changes in material dosing and the related limonene concentration take longer to detect. In both cases where the concentration was altered, it took around 8 min for a change to be detected. This resulted in the limonene concentration in the gas stream taking longer to reach equilibrium. Therefore, the detected limonene concentration in the gas stream required approximately 20 min to reach equilibrium. One potential explanation for these findings is the considerably lower initial concentration of limonene in the DSD-324 material and consequently in the entire compound. In combination with the elevated dilution, this results in a reduced quantity of limonene being analyzed by the SIFT-MS and a subsequent delay in detection. The measurement results also show significantly higher fluctuations than those obtained from homogeneously contaminated materials. This highlights the inhomogeneity, and therefore the varying levels of contamination, in the input streams from open loop collections. This confirms the hypothesis put forward in the introduction. Assuming that homogenization took place in the melt during the extrusion process and subsequently in the gas phase, the fluctuations observed here are smaller than in offline measurements. This illustrates the problem with analyzing small samples randomly taken from a produced batch and then transferring these results to the entire batch.

## 4 Conclusions

This study demonstrates the suitability of SIFT-MS as an effective real-time analytical method for detecting VOCs during the extrusion step of mechanical plastics recycling. By enabling continuous monitoring of the VOC emissions directly from the melt in the degassing step, the method offers information on the chemical quality of recycled materials. The results of the experimental setup demonstrate that the method can identify target components in the gas stream. Furthermore, a strong linear correlation was observed between the concentration of VOCs in the input material and their corresponding concentration and signal intensity in the gas phase. This confirms the quantitative capabilities of the method. However, high VOC concentrations may require substantial gas dilution to prevent depletion of reagent ions and ensure reliable quantification. At the same time, it can be deduced that substances in the ppb range can also be detected. The detectability of less volatile substances can be improved by increasing their diffusion into the gas phase from the melt. Process parameters such as temperature, vacuum level, and dilution rate were shown to significantly influence detection sensitivity, suggesting clear pathways for method optimization.

As a result, SIFT-MS provides a powerful tool for quality assurance in plastic recycling, particularly for applications with chemical safety requirements, such as food contact or cosmetic packaging. With regard to its use for quality control, it was shown that the method is suitable for determining the concentration of volatile target substances in real time. Deviations in concentration at the vacuum dome are detected within seconds, enabling almost continuous process monitoring. Future applications could include integration into the extrusion process control system, for example to respond immediately to quality deviations by stopping the process or changing setup parameters. This would guarantee a significant increase in safety, especially when using recycled materials in sensitive applications.

This feasibility study also provides a foundation for further research for the detection of VOCs in the extrusion process in real time using SIFT-MS. At the same time, it shows potential for optimization with regard to sampling and the measurement setup and parameters. As previously mentioned, future studies should investigate the correlation between the concentrations in the input material and the concentration measured in the gas stream. This is particularly dependent on the diffusion behavior of the VOCs from the melt into the gas phase. The rate of this process depends on the properties of the volatile substances, as well as the parameters of the extrusion process, such as temperature, the free surface area and the pressure in the sampling area.

## Abbreviations

BPR: back pressure regulator
cps: counts per second
FDA: Food and Drug Administration
FS: full scan
GC-MS: gas chromatography–mass spectrometry
HDPE: high-density polyethylene
MFC: mass flow controller
*m*/*z*: mass-to-charge
PCR: postconsume recyclate
PP: polypropylene
ppmV: parts per million by volume
QMS: quadrupole mass spectrometer
sccm: standard cubic centimeters per minute
SIFT-MS: Selected Ion Flow Tube Mass Spectrometry
SIM: selected ion mode
VOC: volatile organic compounds
